# A cadaveric model to evaluate the effect of unloading the medial quadriceps on patellar tracking and patellofemoral joint pressure and stability

**DOI:** 10.1186/s40634-018-0150-8

**Published:** 2018-09-10

**Authors:** Joanna Stephen, Avinash Alva, Punyawan Lumpaopong, Andy Williams, Andrew A. Amis

**Affiliations:** 1 Biomechanics Group, Mechanical Engineering Department, Imperial College London, London, SW7 2AZ UK; 2 Orthopaedic Surgery Department, Imperial College School of Medicine, Chelsea and Westminster Hospital, London, SW10 9NH UK; 3 Fortius Clinic, Fitzhardinge Street, London, W1H 6EQ UK; 4 Musculoskeletal Surgery Group, Imperial College School of Medicine, Charing Cross Hospital, London, W8 6RF UK

**Keywords:** Patellofemoral joint, Vastus medialis, Muscle weakness, Contact pressure, Kinematics

## Abstract

**Background:**

Vastus Medialis Muscles (VMM) damage has been widely identified following patellar dislocation. Rehabilitation programmes have been suggested to strengthen the VMM and reduce clinical symptoms of pain and instability. This controlled laboratory study investigated the hypothesis that reduced Vastus Medialis Obliquus (VMO) and Vastus Medialis Longus (VML) muscle tension would alter patellar tracking, stability and PFJ contact pressures.

**Methods:**

Nine fresh-frozen dissected cadaveric knees were mounted in a rig with the quadriceps and iliotibial band loaded to 205 N. An optical tracking system measured joint kinematics and pressure sensitive film between the patella and trochlea measured PFJ contact pressures. Measurements were repeated for three conditions: 1. With all quadriceps heads and iliotibial band (ITB) loaded; 2. as 1, but with the VMO muscle unloaded and 3. as 1, but with the VMO and VML unloaded. Measurements were also repeated for the three conditions with a 10 N lateral displacement force applied to the patella.

**Results:**

Reduction of VMM tension resulted in significant increases in lateral patellar tilt (2.8°) and translation (4 mm), with elevated lateral and reduced medial joint contact pressures from 0.48 to 0.14 MPa, and reduced patellar stability (all *p* < 0.05).

**Conclusions:**

These findings provide basic scientific rationale to support the role of quadriceps strengthening to resist patellar lateral maltracking and rebalance the articular contact pressure away from the lateral facet in patients with normal patellofemoral joint anatomy.

## Background

Patellofemoral joint (PFJ) complaints account for 25–40% of all knee related problems presenting to sports medicine centres and represent the most common sporting knee injury in the under-50 population (Almeida et al. 1999; Taunton et al. 2002). Insufficiency of the vastus medialis muscles (VMM), particularly the Vastus Medialis Obliquus (VMO), may contribute to the initiation or aggravation of patellar pain and instability, due to the reduction in the medial force acting on the patella (Fulkerson 2002). The VMM has historically been reported to subdivide into two components: a proximal portion referred to as the vastus medialis longus (VML), and a distal portion referred to as the vastus medialis oblique (VMO) (Lieb and Perry 1968), with the VML postulated to contribute directly to knee extension, whilst the VMO had its role as a medial patellar stabilizer (Lieb and Perry 1968). Innervation patterns of the muscles have been investigated with some studies reporting differing spinal segment innervation patterns going to VMO and VML, suggesting they function independently. More recently however, this has been disputed (Skinner and Adds 2012). The anatomy of the vastus medialis remains controversial, with some studies reporting a consistent band of adipose fat to separate the VMO and the vastus medialis longus (VML) (Scharf et al. 1985; Thiranagama 1990), whilst other studies have found the presence of this to be inconsistent (Skinner and Adds 2012; Hubbard et al. 1997). EMG investigations have found no functional differences exist between the VMO and VML, suggesting they may not be separate muscle entities (Hubbard and Opersteny 2002). Overall there is a lack of agreement to support whether the VMO is an anatomically separate structure to the VML, and so for the purpose of this study both are considered.

VMO atrophy measured using Magnetic Resonance Imaging (MRI) scans and reduced electromyography (EMG) magnitude and timing have been identified in patients suffering patellofemoral pain (Pattyn et al. 2011; Souza 1991). Ultrasound studies have reported the pennation angle of the VMO can be increased with quadriceps strengthening programmes (Khoshkhoo et al. 2016). Tearing of the VMM has been reported following traumatic patellar dislocation (Ahmad et al. 2000; Hunter et al. 1983). However, the role of the VMM in patellar tracking and stability remains the subject of some debate (Powers 1998; Mihalko et al. 2007), with literature often focusing on the importance of core stabilisers (gluteals, abdominals) rather than the local stabilisers (quadriceps) (Bolgla 2008; Powers 2003). Furthermore, dynamic structures have been suggested to have less of a role in patellar stability than local ligaments and bony anatomy (Senavongse and Amis 2005).

Prior studies have explored the effect of altering VMO tension on patellar tracking (Goh et al. 1995), however the role of the quadriceps in providing mechanical stability to the patella (patellar resistance to lateral displacement) has not previously been closely examined. Such an investigation would help support the role of quadriceps rehabilitation programmes and conservative management for patients post patellar dislocation. Prior cadaveric work examining the effect of altered VMO loading has also not examined contact mechanics and joint kinematics simultaneously, through full range of knee flexion (Goh et al. 1995; Elias et al. 2009; Lee et al. 2002; Wünschel et al. 2011). It is widely hypothesised that abnormal patellar tracking causes increased contact stresses and subsequent cartilage wear, and having contact pressure data alongside tracking data would help support or negate this theory (Dye et al. 1998). Furthermore, no work has so far examined the effect of deactivation of the VMM (the VMO plus the vastus medialis longus – VML) which may occur during traumatic patellar dislocation when the fibres are torn. Based on prior research examining VMO weakness and current knowledge we hypothesised that deactivation of the VMM would result in increased lateral patellar tracking, increased lateral cartilage contact pressure and reduced patellar stability.

## Methods

### Specimen preparation

Four male and five female right-sided, fresh-frozen cadaveric knees of mean age 64 years (range: 48–92), with no history of knee surgery or disease were obtained from a tissue bank following approval from the local research ethics committee. Specimens had approximately 150 mm of tibia and 200 mm of femur and were stored at − 20 °C in polyethylene bags. MRI scans were taken of all knees and Tibial Tuberosity - Trochlear Groove (TT-TG) distance (Dejour et al. 1994) (range: 11 mm–19 mm), Sulcus Angle (Davies et al. 2000) (range: 131°–144°) and Insall-Salvati ratio (Grelsamer and Meadows 1992) (range: 0.8–1.1) were found to be within reported normal limits for all specimens.

Skin and subcutaneous fat were removed; the iliotibial band (ITB), deep fascia and retinacula were preserved. The fibular head was fixed to the tibia with two bone screws. Intramedullary rods were cemented into the femur and tibia. The patella was measured and the proximal-distal centre of the median patellar ridge determined: this was termed the ‘functional centre point’ (CP) (Merican et al. 2009). In preparation for patellar stability testing, a 10 mm diameter cavity was drilled 10 mm deep from the anterior surface of the patella (the patellar cartilaginous layer was not broken) at the CP and a polyethylene socket secured into the hole with four 13 mm long screws.

The quadriceps were divided into six components: Rectus Femoris (RF); Vastus Intermedius (VI); Vastus Medialis Longus (VML); Vastus Medialis Obliquus (VMO); Vastus Lateralis Longus (VLL) and Vastus Lateralis Obliquus (VLO). Fabric was sutured to each muscle and the ITB, and this augment was securely attached to a cable. The muscles and ITB were tensed in accordance with directions and cross-sectional areas previously described (Farahmand et al. 1998a); a total load of 205 N was applied (Farahmand et al. 1998b; Merican and Amis 2009). The ITB contributes up to 10%–20% of the restraint to lateral patellar motion (Christoforakis et al. 2006; Senavongse et al. 2003), prior work suggests the ITB should be loaded when studying patellar kinematics (Merican and Amis 2009). It was therefore loaded with 30 N tension in accordance with prior work and its proportion to a quadriceps tension of 175 N (Merican and Amis 2009; Kwak et al. 2000). The applied loads correspond to an open chain knee extension motion; higher loads were not applied in order to avoid damaging local soft tissues (Stephen et al. 2013).

**Fig. 1:**
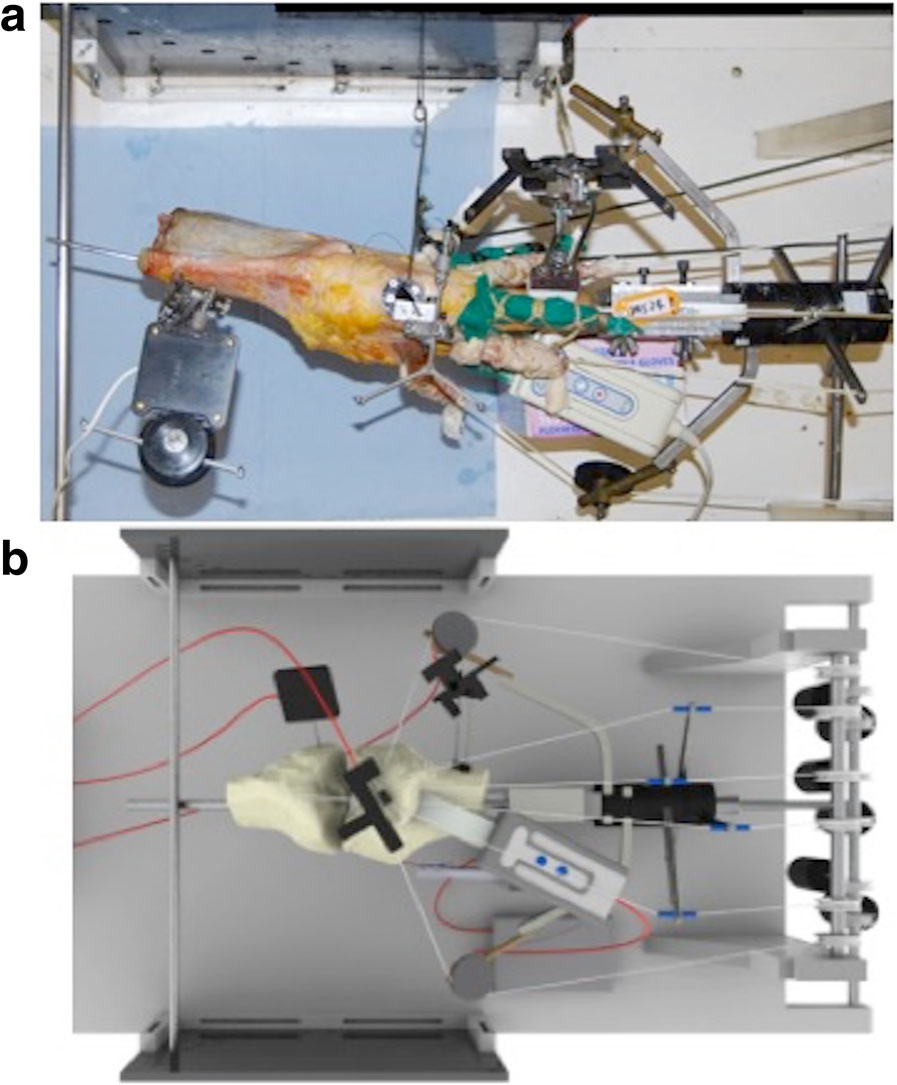
**a**, **b**: photograph and schematic of the test rig, showing multiple loading cables attached to the muscles and ITB, plus optical trackers mounted on the femur, tibia and patella

The knee was mounted in the test rig with the anterior aspect facing upwards and the posterior femoral condylar axis horizontal (Fig. 1). A transverse bar mounted across the anterior aspect of the tibial intramedullary rod held the tibia at progressive 10° increments of knee flexion, permitting secondary tibial movements, but keeping the femur fixed. This also enabled the flexion angle to be measured during testing.

### Optical tracking

Active optical trackers (Traxtal Technologies, Toronto, Canada) were attached to the patella, femur and tibia using custom-made blocks screwed securely to the bones, preventing any motion between the tracker and the bone. A Polaris optical tracking system with Toolviewer software (Northern Digital Incorporated, Waterloo, Canada) measured patellar kinematics. Metal fiducial markers attached to the patella, femur and tibia were digitized using a Traxtal probe to build a local co-ordinate system of each bone (Stephen et al. 2013). The Polaris system had a known overall root mean square distance error of 0.35 mm for a single marker (Wiles et al. 2004). Patellar motion was described in relation to the femur by a standard convention (Merican and Amis 2009; Bull et al. 2002).

### Contact pressure measurement

A Tekscan 5051 sensor (Tekscan, I-Scan™, Boston MA, USA) measured PFJ contact pressures from 0° to 90° knee flexion. The saturation pressure of the sensor was 3.48 MPa, and the sensor was 0.1 mm thick, and 55.9 mm by 55.9 mm. Following equilibration and calibration, in accordance with manufacturer guidelines, it was inserted through a superior incision in the patellofemoral pouch. The sensor was secured between the patella and trochlea, covering both surface areas (Stephen et al. 2013). Two sutures attached to the distal corners of the sensors were stitched to the local soft tissue to prevent movement of the sensor during knee extension. A 1 mm rod inserted through a hole drilled in the patella imprinted on the Tekscan film during each recording. The hole was referenced to the median patellar ridge to differentiate medial and lateral facets.

### Stability testing

Patellar mechanical stability was considered as patellar resistance to lateral displacement and measured as the lateral translation of the centre of the patella induced by applying a 10 N lateral displacement force to the patella using a hook, attached to a weight over a pulley, which fitted into the polyethylene socket at the centre of the patella, pulling it laterally. The application of the lateral load did not inhibit the natural rotations of the patella, enabling all six degrees of freedom of patellar motion to be assessed. Patellar motion was described in relation to the femur by a standard convention (Merican and Amis 2009; Bull et al. 2002).

### Experimental protocol

Knees underwent ten ‘conditioning cycles’ from 0° to 90° flexion, in order to minimise hysteresis. Kinematic and pressure data were collected at 0°, 10°, 20°, 30°, 60° and 90° of knee flexion. The order of testing was randomised to avoid bias. When the VMO and VML were unloaded, the load which would have been applied to them was redistributed amongst the other quadriceps portions in proportion to their cross sectional area. In accordance with prior findings using the same loading set-up, the ITB was loaded with 30 N throughout testing (Merican and Amis 2009), therefore a constant 205 N was applied. Measurements were taken for each condition both with and without an external lateral 10 N displacing force applied. The 10 N load was determined based on prior cadaveric studies investigating patellar stability (Nomura et al. 2000; Stephen et al. 2015).

### Statistical analysis

Custom written Matlab scripts (MATLAB 8.0, The MathWorks Inc., Natick, MA) calculated mean and peak contact pressures and patellar motion from the raw data produced by the Iscan and Toolviewer software. A power calculation determined a sample size of nine necessary to detect a significant PFJ contact pressure changes of 10% with 80% power and 95% confidence (Elias et al. 2009). The dependent variables were: mean and peak, medial and lateral facet articular contact pressures, and patellar translation and tilt. Data was analyzed in SPSS (IBM Corp. Version 20.0. Armonk, NY). A Kolmogorov–Smirnov test confirmed that the data sets were normally distributed.

Two different effects were investigated: muscle forces and lateral force (patellar stability). Two-way repeated measures analysis of variance (RM ANOVA) were performed with the three muscle load effects (normal loading, no VMO and no VMO or VML) and flexion angle (0°, 10°, 20°, 30°, 60° and 90°) for each of the dependent variables examined. When muscle load significantly influenced the output, post-hoc paired t-tests with Bonferroni correction were applied comparing the normal loaded knee with each of the other conditions at individual flexion angles.

## Results

### Effect of altering muscle loading pattern

**Fig. 2:**
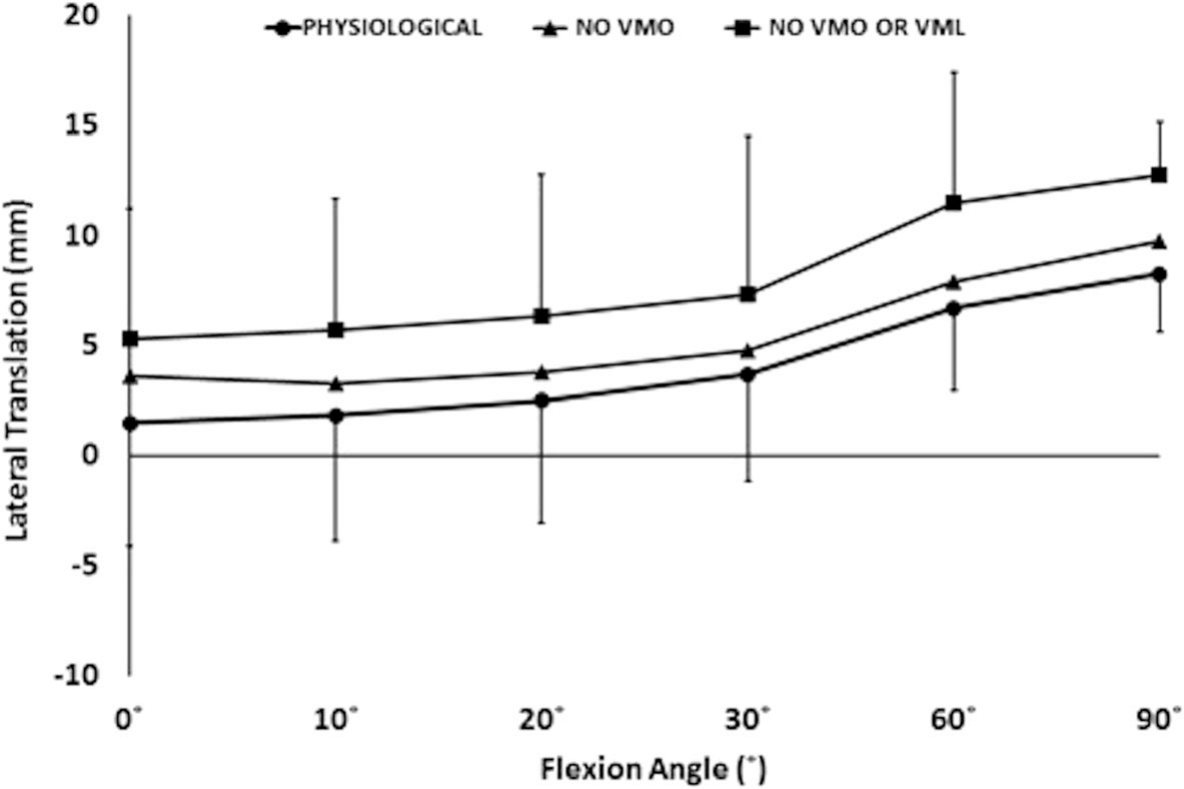
Patellar lateral translation (mm) across knee flexion (mean +/- SD; n=9)

**Fig. 3:**
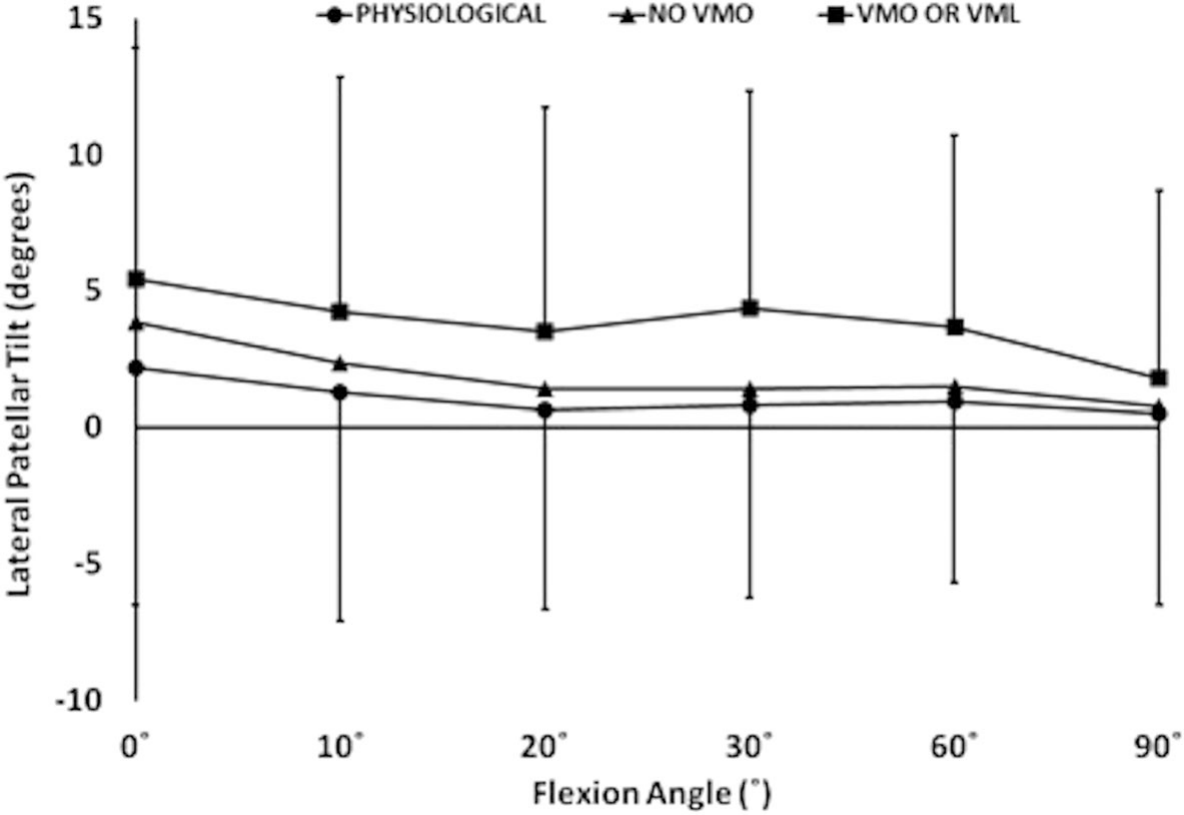
Patellar lateral tilt (°) across knee flexion (mean +/- SD; n=9)

**Fig. 4:**
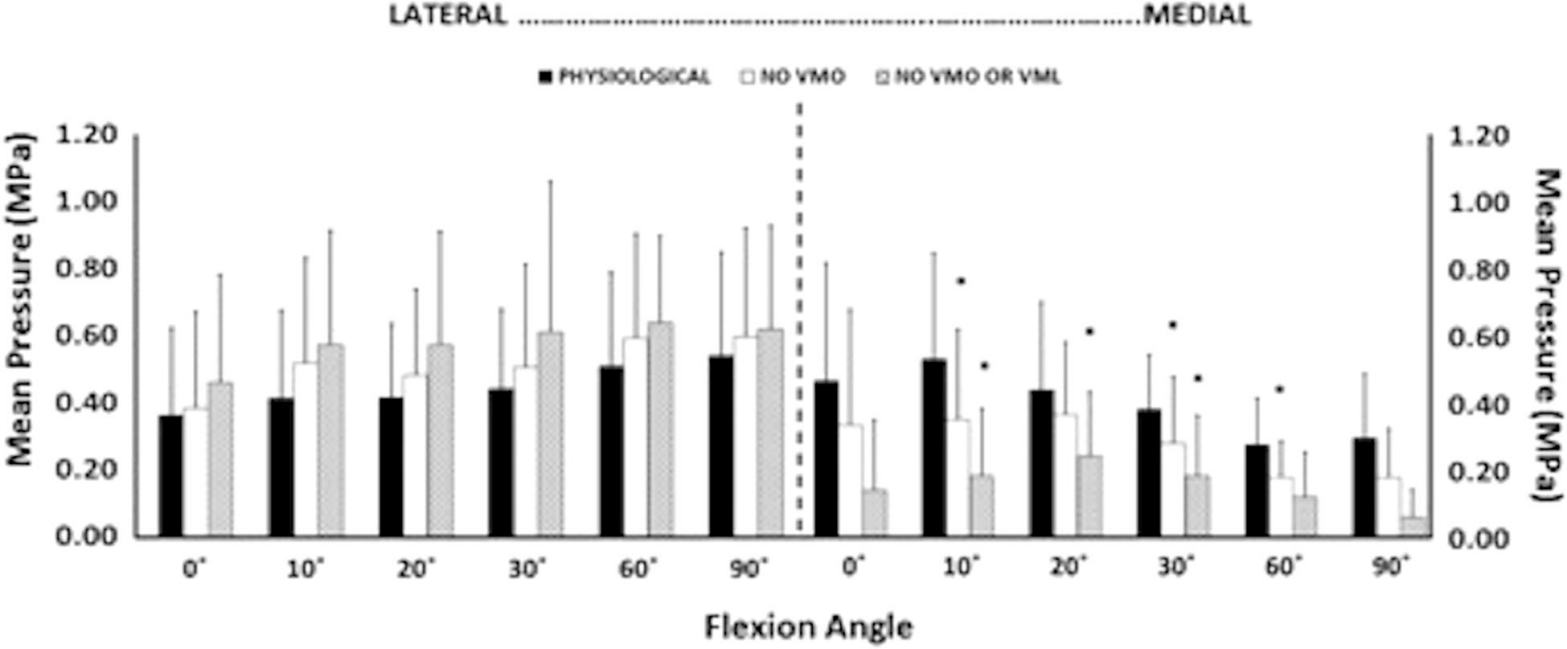
Mean contact pressures for lateral and medial facets, across 0 to 90° knee flexion, showing efffects of relaxing the VMM. * *p* < 0.05

**Fig. 5:**
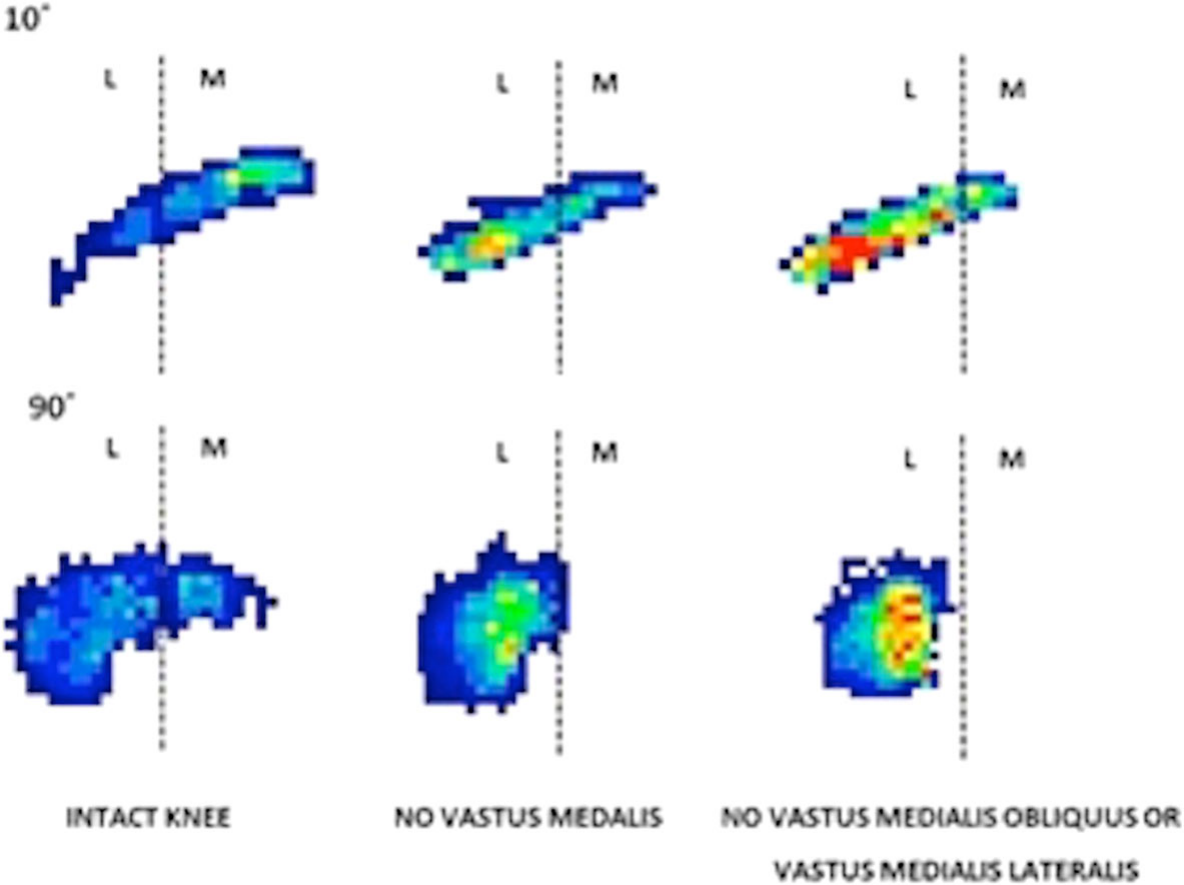
Tekscan pressure maps for two knees, one at 10° flexion, the second at 90° flexion. Reduced tension in the VMM led to increased pressure on the lateral facet (Dark blue: 0.2 MPa; green: 0.5 MPa; yellow: 1.0 MPa; red: 1.5 MPa)

**Fig. 6:**
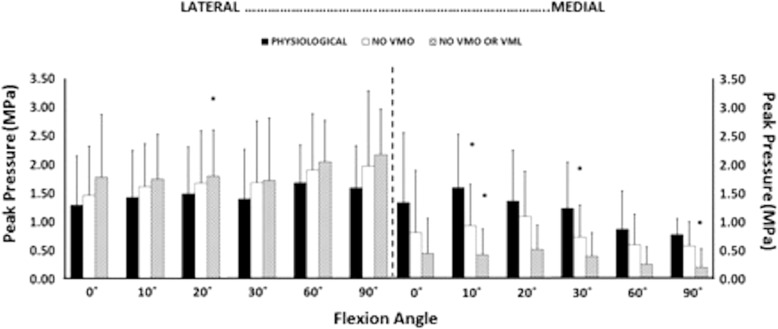
Peak contact pressure on each of the meadial and lateral facets of the joint, across 0 to 90^o^ knee flexion, showing the effects of relaxing the VMM; * *P* < 0.05

Removal of the load applied to the VMO, and the VMO and VML muscles together, resulted in an overall increase in lateral motion of the patella throughout flexion. With the VMO and VML unloaded, lateral patellar translation (or ‘shift’) and tilt increased by a mean of 4 mm and 2.8° (Figs. 2 and 3); mean medial articular joint pressure reduced from 0.48 to 0.14 MPa at 0° (RM ANOVA all: *P* < 0.05) (Fig. 4). There was a significant increase in mean and peak lateral contact pressure (both *P* < 0.05) and a significant decrease in peak medial contact pressure (*P* < 0.05) (Figs. 5 and 6). Flexion angle had a significant effect on mean and peak medial contact pressures, with muscle loading having a greater effect in early flexion, before the patella engaged with the trochlear groove (all: *P* < 0.05). No significant effect of flexion angle was found with any of the other variables (all: *p* > 0.05). Figures 2, 3, 4 and 5 highlight post-hoc test findings.

### Effect of muscle load and lateral displacing force

**Fig. 7:**
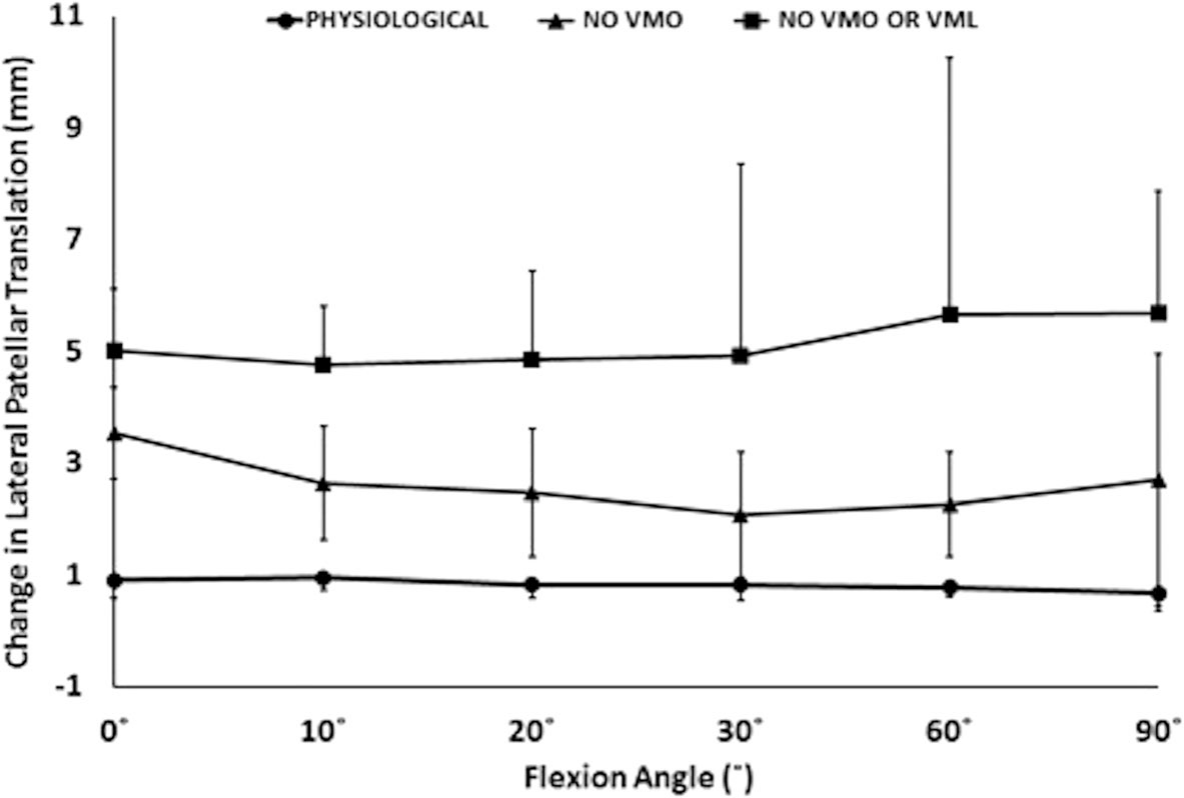
Change of patellar lateral translation in response to a 10 N lateral displacing force, showing increased effect after relaxation of the VMM

**Fig. 8:**
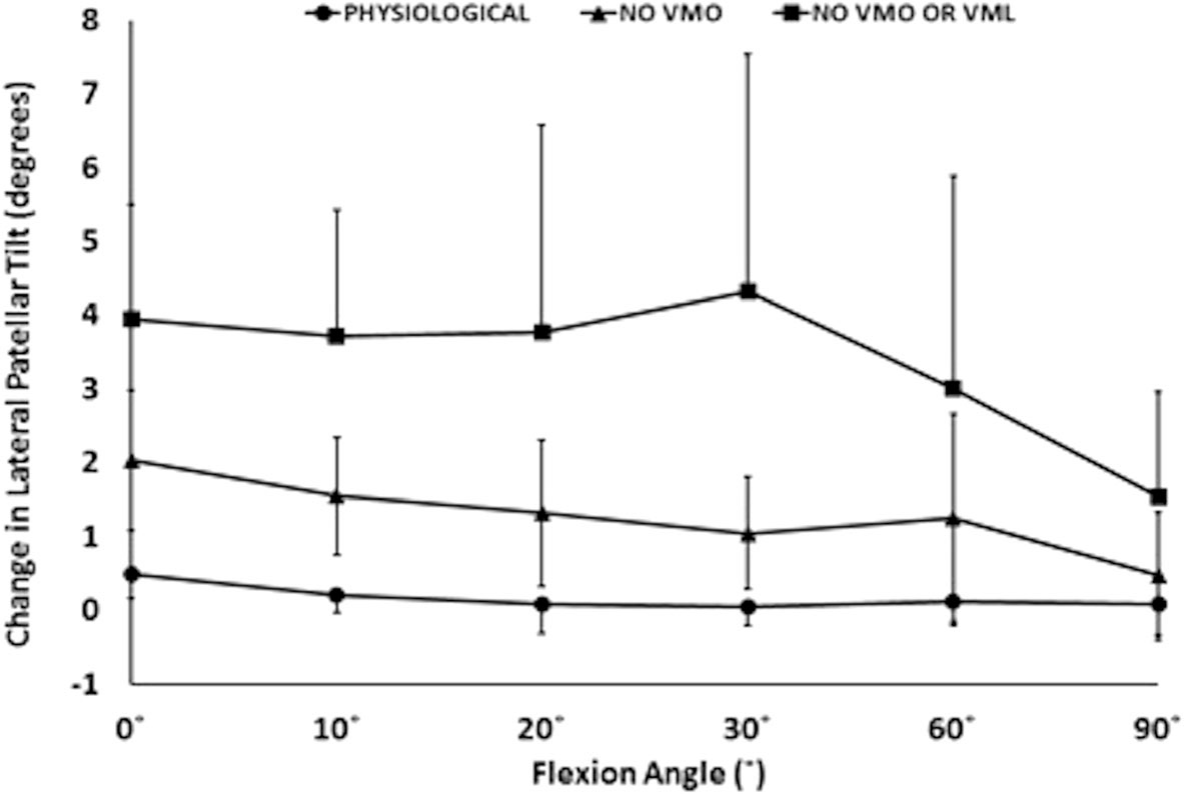
Change of patellar lateral tilt in response to a 10 N lateral displacing force, showing increased effect when the VMM had been relaxed

**Fig. 9:**
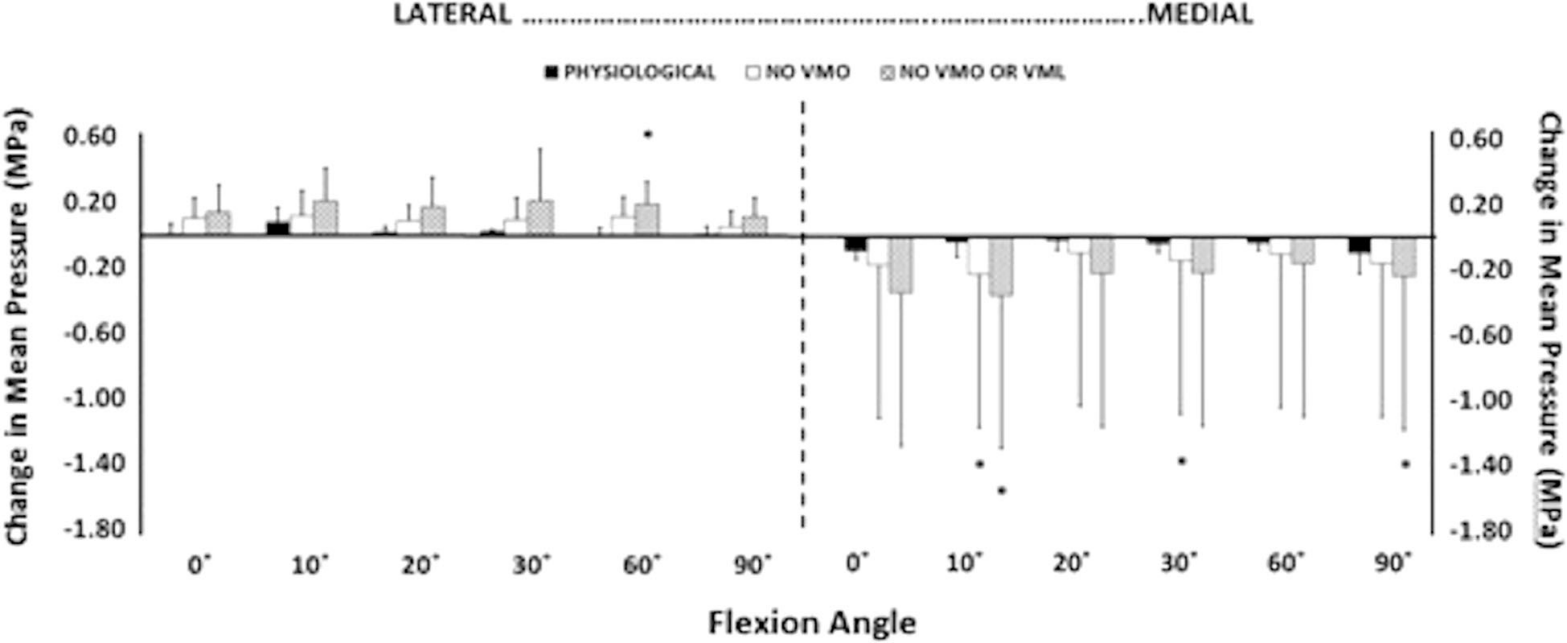
Change of mean patellofemoral contact pressure, across knee flexion, caused by imposing a 10 N lateral displacing force to the patella

**Fig. 10:**
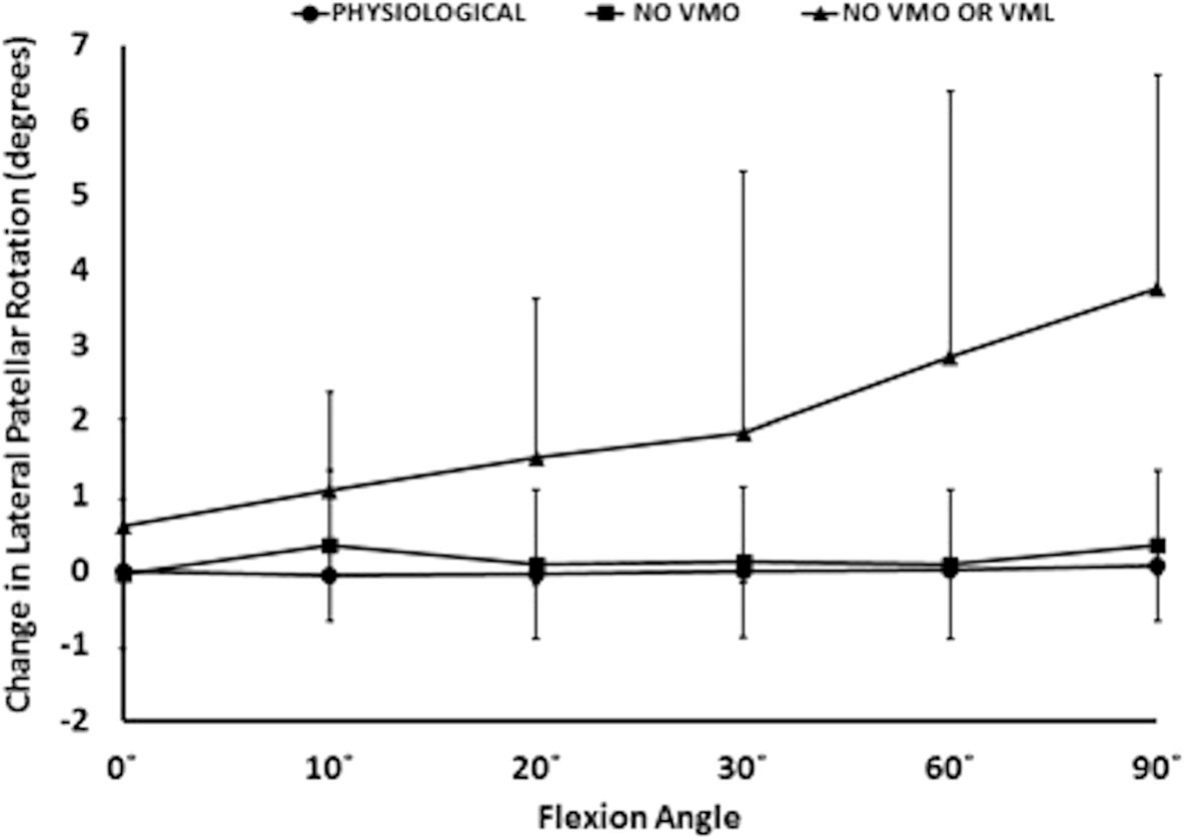
Relaxation of the VMM led to increased patellar lateral rotation; that is: the distal pole moved relatively lateral

The effect of the 10 N laterally directed force increased significantly as the VMO and VMO and VML were unloaded. In the normally loaded knee the patella was 1.5 mm lateral in full extension (Fig. 2). The 10 N lateral force increased lateral patellar translation by 0.9 mm (Fig. 6) to 2.4 mm lateral. Unloading the VMO and VML resulted in the patella lateralising 3.8 mm, from 1.5 to 5.3 mm lateral (Fig. 2), which was increased a further 1.2 to 6.5 mm lateral following the application of a 10 N lateral load. Therefore the combined effect of relaxed VMM and 10 N lateral load equalled an extra 5 mm subluxation (3.8 mm + 1.2 mm) (Fig. 7). There was a significant effect of the lateral load application on all dependent variables investigated (all: *P* < 0.05): patellar translation and tilt (Figs. 7 and 8), and mean and peak medial and lateral contact pressures (Fig. 9). The increase in lateral patellar tilt was dependent on knee flexion, with the effect of altering muscle load reducing as the knee was flexed (*P* < 0.05). No other variables were affected by angle of knee flexion (*P* > 0.05). It was also found that reduced VMM tension led to increased patellar lateral rotation (That is: the distal pole of the patella moved relatively leterally (Fig. 10)).

## Discussion

The most important finding of this work was that removing tension from the VMO and VML muscles resulted in increased lateral displacement of the patella with significant increases in lateral patellar translation, tilt and mean and peak lateral PFJ contact pressures alongside reductions in mean and peak medial contact pressures. The effect of a 10 N laterally-directed displacement force on the patella was significantly increased following medial quadriceps unloading, highlighting a reduction in patellar stability. The results emphasise that a lack of VMM function increases lateral compartment PFJ pressure throughout full range of knee flexion, permitting a more lateralised position of the patella and increased vulnerability to dislocation. Current findings support the role of VMO in patellar stability and are similar to those highlighting the stabilising role of the MPFL (Senavongse and Amis 2005; Stephen et al. 2013). VMO injury has been described alongside MPFL injury in patients with patellofemoral dislocation (Pattyn et al. 2011; Zhang et al. 2018) and this, considered alongside current findings, provides a rationale for the implementation of quadriceps rehabilitation programmes in patellar dislocation patients.

Study limitations include the use of elderly cadaveric knees with normal PFJ geometry, preventing direct extrapolation of results to patient populations suffering patellar dislocation. The Tekscan was inserted using a method intended to minimise disruption to the retinacula but it required the opening of the superior PFJ capsule and lifting / dissection of VI from the femur, which may have influenced patellar behaviour. However these effects were constant throughout all testing, and a proximal approach to access the PFJ was the least invasive method to permit insertion of the Tekscan film whilst leaving local soft tissues intact. Muscles were loaded in their anatomical directions, as previously determined (Farahmand et al. 1998a) but tensions were lower than those reported in vivo and constant through knee flexion and therefore not directly comparable to weight-bearing activity (Cohen et al. 2001). The complete relaxation of the VMM in this study is probably more extreme than occurs in-vivo, and so the results are likely to be showing the largest changes which may occur clinically. In life, there will be a spectrum of muscle inhibition and damage to surrounding soft tissues, but we do not know what is most representative when simulating the abnormal knee in-vitro.

Prior work has found an effect on patellofemoral contact pressure and patellar tracking from altering quadriceps loading (Goh et al. 1995; Elias et al. 2009; Lee et al. 2002; Powers et al. 1998; Lieb and Perry 1968). However this is the first study to examine these effects on medial and lateral contact pressures and patellar motion simultaneously, through full range of knee motion without having damaged either medial or lateral retinacula during pressure film insertion. This is relevant to help support the theory that patellar maltracking alters joint contact stresses which in turn may result in joint degeneration and possibly pain. Tearing, inhibition and atrophy of the VMO have been identified clinically in patients with PFJ pathology (Cowan et al. 2003; Crossley et al. 2002; Sallay et al. 1996). The findings highlight that removal of VMO tension (as may happen clinically as a result of trauma or pain) results in lateralised PFJ contact pressures and patellar motion throughout full knee flexion. PFJ patients commonly report symptoms during activities necessitating quadriceps contraction such as ascending and descending stairs and standing up. We could therefore hypothesise that lack of VMO function results in elevated contact pressures on the lateral facet of the PFJ as a consequence of reduced patellar - trochlear contact area. Future clinical studies could examine this relationship in order to obtain data to support the implementation of quadriceps strengthening programmes and modalities such as muscle stimulators to improve quadriceps activation in PFJ instability populations.

Earlier work examining patellofemoral stability has identified the medial patellofemoral ligament (MPFL) to be the most important passive stabiliser through early knee flexion, with bony geometry the most critical factor in deeper flexion (Senavongse and Amis 2005). However the VMO has generally been considered to have a role, albeit a lesser role, in PFJ stability. Previous work (Senavongse et al. 2003) reported increases of up to 2 mm in lateral patellar translation and 1° lateral tilt following MPFL transection at 30° knee flexion. This study found a similar effect of VMO unloading with increased lateral translation and tilt up to 2.1 mm and 1.7° permitted respectively throughout the range of flexion. The relationship of the VMO and MPFL has not been consistently defined (Bicos et al. 2007) but it has been suggested that the fibres of each inter-link with one another (Smirk and Morris 2003), with both structures contributing to patellar stability. Thus relaxation of the VMO in the present study may have had a secondary effect of reducing MPFL fibre tension and thus efficiency. Current study findings support the role of the medial vastus fibres as stabilisers, since removal of applied muscle tension and application of a 10 N lateral load resulted in significantly increased lateral patellar motion and lateral PFJ contact pressures and reduced medial PFJ pressures. These findings provide a basic science rationale to support the implementation of a VMO and VML based strengthening programme for PFJ dislocation patients, which would be less invasive than MPFL reconstruction.

## Conclusions

The present study outcomes highlight the significant contribution of the medial vasti muscles to PFJ tracking and stability. Loss of VMO and VMO + VML function resulted in significantly increased lateral patellar tracking, with increased lateral PFJ contact pressures and reduced medial joint contact pressures and PFJ stability. VMO tearing and atrophy have been described in patients suffering patellofemoral dislocation and this, taken together with current findings, suggests there may be a role for quadriceps strengthening rehabilitation programmes.
